# Supplementary material for: Long-Term Evolution of Activities of Daily Life (ADLs) in Critically Ill COVID-19 Patients, a Case Series
Source: Healthcare (Basel). 2023 Feb 23;11(5):650. doi: 10.3390/healthcare11050650 (PMC10001119; doi:10.3390/healthcare11050650)
Supplement: Supplementary file 1 [file healthcare-11-00650-s001.zip › healthcare-2163311-supplementary.pdf]

healthcare

MDPI

Table S1. Barthel index.

Activity	Score
**FEEDING**	
0 = unable<br>5 = needs help cutting, spreading butter, etc., or requires modified diet<br>10 = independent	
**BATHING**	
0 = dependent<br>5 = independent (or in shower)	
**GROOMING**	
0 = needs to help with personal care<br>5 = independent face/hair/teeth/shaving (implements provided)	
**DRESSING**	
0 = dependent<br>5 = needs help but can do about half unaided<br>10 = independent (including buttons, zips, laces, etc.)	
**BOWELS**	
0 = incontinent (or needs to be given enemas)<br>5 = occasional accident<br>10 = continent	
**BLADDER**	
0 = incontinent, or catheterized and unable to manage alone<br>5 = occasional accident<br>10 = continent	
**TOILET USE**	
0 = dependent<br>5 = needs some help, but can do something alone<br>10 = independent (on and off, dressing, wiping)	
**TRANSFERS (BED TO CHAIR AND BACK)**	
0 = unable, no sitting balance<br>5 = major help (one or two people, physical), can sit<br>10 = minor help (verbal or physical)<br>15 = independent	
**MOBILITY (ON LEVEL SURFACES)**	
0 = immobile or < 50 yards<br>5 = wheelchair independent, including corners, > 50 yards<br>10 = walks with help of one person (verbal or physical) > 50 yards<br>15= independent (but may use any aid; for example, stick) > 50 yards	
**STAIRS**	
0 = unable<br>5 = needs help (verbal, physical, carrying aid)<br>10 = independent	
**TOTAL (0-100)**	

The BI measures ten essential self-care and physical dependency aspects, rating each ADL's element. A score of 100 denotes normality, lower scores indicate increasing disability [33].

Table S2. Karnofsky Performance Status scale.

Able to carry on normal activity and to work; no special care needed.	100	Normal no complaints; no evidence of disease.
	90	Able to carry on normal activity; minor signs or symptoms of disease.
	80	Normal activity with effort; some signs or symptoms of disease.
Unable to work; able to live at home and care for most personal needs; varying amount of assistance needed.	70	Cares for self; unable to carry on normal activity or to do active work.
	60	Requires occasional assistance, but is able to care for most of his personal needs.
	50	Requires considerable assistance and frequent medical care.
Unable to care for self; requires equivalent of institutional or hospital care; disease may be progressing rapidly.	40	Disable; requires special care and assistance.
	30	Severely disabled; hospital admission is indicated although death not imminent.
	20	Very sick; hospital admission necessary; active supportive treatment necessary.
	10	Moribund; fatal processes progressing rapidly.
	0	Dead

The KPS is helpful to assess functional impairment, measuring a patient's general performance status or ability to carry out activities of daily living. It classifies patients according to their functional impairment and allows comparisons between patients : the lower the KPS score, the worse the survival for most severe diseases. Nowadays, KPS is used to predict the outcome of numerous other pathologies and clinical conditions besides cancer [43]. It ranges from 100 (no restrictions) to 0 points (death).

Table S3. Normality tests.

Case Processing Summary						
	Cases					
	Valid		Missing		Total	
	N	Percent	N	Percent	N	Percent
Barthel on discarge of hospital	17	68,0%	8	32,0%	25	100,0%
Barthel 1 years after discarge	17	68,0%	8	32,0%	25	100,0%

		Mean	95% CI for Mean		5% Trimmed Mean	Median	Variance	SD	Min	Max	Range	IQR
			Lower Bound	Upper Bound								
Barthel on discarge of hospital	Statistic	74,12	59,80	88,44	76,52	80,00	775,735	27,852	5	100	95	38
	Std. Error	6,755										
Barthel 1 years after discarge	Statistic	100,00	100,00	100,00	100,00	100,00	0,000	0,000	100	100	0	0
	Std. Error	0,000										

Extreme Values											
		1		2		3		4		5	
		Highest	Lowest	Highest	Lowest	Highest	Lowest	Highest	Lowest	Highest	Lowest
Barthel on discarge of hospital	Case Number	6	1	17	2	19	13	20	15	9	8
	Value	100	5	100	20	100	55	100	60	95	60
Barthel 1 years after discarge	Case Number	1	20	2	19	3	17	5	16	6	15
	Value	100	100	100	100	100	100	100	100	100[a]	100[b]

a. Only a partial list of cases with the value 100 are shown in the table of upper extremes.
b. Only a partial list of cases with the value 100 are shown in the table of lower extremes.

Tests of Normality						
	Kolmogorov-Smirnov[a]			Shapiro-Wilk		
	Statistic	df	Sig.	Statistic	df	Sig.
Barthel on discarge of hospital	0,186	17	0,119	0,843	17	0,009
Barthel 1 years after discarge		17			17	

a. Lilliefors Significance Correction
